# The Cattle Plague and Diseased Meat, in Their Relations with the Public Health

**Published:** 1858-01

**Authors:** 


ART. III.—*The Cattle Plague and Diseased Meat, in their Relations with the Public Health, and with the Interests of Agriculture. A Letter to the Rt. Hon. Sir George Grey, Bart., G.C.B., Secretary of State for the Home Department.* By JOSEPH SAMPSON GAMGEE, Staff-Surgeon of the First Class, and Principal Medical Officer of the British-Italian Legion during the last war, etc., etc. London, 1857.

*A Second Letter on the same subject by the same author.*

IN these "bold and admirable" letters Mr. Gamgee adds the distinction of a very decided and efficient sanitary champion to his previously well-earned character as a zealous and able morbid anatomist and practical surgeon. Having the rare advantage of veterinary experience, it is not surprising that his intelligent and indefatigable energy should have led him to attack an evil which had long disgusted him, and to which a committee of Medical Officers of Health had already formally, but in vain, directed the attention of the London public.

The keen determination with which he conducted the inquiry, and his straight-forward and impressive, although scrupulously courteous manner of presenting and discussing its results, seem, at last, to have stirred up a very natural and wholesome sensation among the parties most concerned. We hope that the alarm thus happily excited by Mr. Gamgee's hue-and-cry may not be allowed to subside until it has led at least to a general understanding of the nature and extent of the apprehended mischief; and to the consequent establishment of the permanently vital public opinion and individual watchfulness which, as has been well said, must, after all, afford the best security against abuses of every kind.

In the mean time the rumors of "hog-cholera" in some parts of our own country, and of "milk-sickness" in others—to say nothing of the still-fed and strychnianized swine and cattle—and the demand and high price obtained for animal food in our large cities, admonish us only too clearly of our own liabilities to similar pests. In conning the pages of our trans-Atlantic pamphleteer, let us not imagine that his revolting pictures of diseased and decaying carcases, slipped calves and equivocal sausages, are native only to the shambles and market-stalls which he has explored and so effectually exposed.

For the purpose of facilitating the exposition of his facts, Mr. Gamgee arranges them in the following four categories:—"1st. *Statement of the existing evil.* 2d. *The inefficiency of existing laws.* 3d. *Statement of impending danger.* 4th. *An exposition of the principles on which the urgently requred legislation should be based.*"

It is unnecessary for us to dwell upon many portions of these different categories, as they are chiefly of local interest only; a few graphic passages, as we read on, may afford us sufficiently useful hints in regard to what might be looked for nearer home.

"FIRSTLY—STATEMENT OF THE EXISTING EVIL.

"The existing evil is multiple. *a.* Public sale of diseased beasts with healthy ones, in consequence of inadequate inspection of live markets and cow-houses. *b.* Inefficient inspection of slaughter-houses and dead-meat markets. *c.* Insufficient state of knowledge on cattle diseases, due, in great measure, to non-observance of the very wise regulations framed by the founders of the Royal Veterinary College of London.

"*a. Public sale of diseased beasts with healthy, in consequence of inadequate inspection of live markets and cow-houses.* It is a publicly notorious fact, repeatedly verified by my brother, that diseased beasts, in very considerable numbers, are sold in the New Cattle Market at Islington, which I inspected on Monday morning the 16th inst. The live beasts were generally extremely well-conditioned and thorougly sound; but standing among them were three diseased beasts. One of these was emaciated and hide-bound, with abscesses in various parts of the body, particularly over the region of the head and neck.

From the clinical observations I made on diseased cattle nine years ago, I believe this case was most probably one of pyæmia following typhoid fever. A second beast was in ill health, viz. thin and feverish, but I could not make a precise diagnosis. The third diseased beast was a fat one: it was lying down, moaning, looking round anxiously at its flanks; pulse 110; respiration 45; pleuro-pneumonia.

"On Friday, the 20th inst., I several times visited the Islington Market, and found in it many diseased beasts. The most remarkable example was a row of twenty-one very small and very old and emaciated cows; several of them bore unmistakable signs of old disease,—one of them was moribund; it was standing in the throng, leaning almost its whole weight on the beast near it, striking out its head, panting for breath at the rate of forty times per minute, emitting large volumes of hot vapor from the lungs; its eyes were fixed and staring in the lean and deepened sockets; in the arteries of the extremities the pulse had ceased to beat; and out of two large ulcers the hinder extremities of the hip-bones protruded through the skin, which seemed artificially stretched over and bound down to a lifeless skeleton. From numerous inquiries in the market, I learned that such a state of things is by no means unfrequent. In reply to my inquiries, an official in the administrative department made the following statement:—'It is notorious about diseased beasts in the market: never a market without them; often beasts are disgraceful to look at,—certainly unfit for human food: could not say why the inspector did not seize them.'"

After some further remarks upon the inadequate inspection of the cattle markets, and upon the number of cows becoming diseased in the London cow-houses and sent to the cattle market or to the slaughter-houses of the metropolis, he describes his observations in the "slaughter-houses and dead-meat markets."

"On Monday, the 16th inst., I inspected one of the slaughter-houses at the New Islington Cattle Market. In it I saw five carcases—three of oxen, two of sheep. One of the latter was of magnificent shape and condition, so far as fat was concerned, but the whole carcase had a uniform dusky red color, evidently the result of general infiltration with bloody serosity. The carcase having been trimmed and completely dressed for the butcher, I had no means of inspecting the viscera. Two of the oxen were much emaciated, and had apparently died from typhus or typhoid fever; they presented numerous bloody extravasations in the subcutaneous, inter-muscular, and sub-pleural cellular tissues. I should have required to see the viscera, in order to state accurately the nature of the disease, but they had been removed. The third ox was large, moderately fat; pleuro-pneumonic. I carried away the lungs of this beast; they were infiltrated with solidified plastic matter in almost their whole extent; so that, whereas their average weight should have been about eight pounds, it was twenty-seven pounds. The disease was in its acute stage. Although the carcase had been very skilfully trimmed and dressed, the flesh in the walls of the chest and abdomen bore unmistakable marks of disease. The slaughter-man stated that these carcases would be conveyed to the city markets, where they would be *sold as food.* In his opinion those carcases were not diseased, nor would they be considered such by the city meat-inspectors. He even maintained that the lungs were not diseased; he said they only contained congealed healthy blood!

"On Sunday, the 23d inst., at 5 P.M., I inspected the same slaughter-house, where several men were busily at work. Twelve very fair recently-dressed carcases were hung up—two of sheep, ten of oxen; additionally three diseased carcases, two of which were dressed but quite warm; a third was in process of *trimming.* These three were very old, extraordinarily lean cows, destitute of the least particle of fat; the flesh was pale, nearly white, extensively ecchymosed, the cellular tissue inflated with gas; in the buttocks of the beast which was being trimmed were huge masses of putrid, bloody, and disintegrated muscle,—the whole appearances were those of advanced typhoid disease. From two of these cows two calves, nearly at full period, had been removed; they were now hung up, and would, from all I have seen in Newgate Market, and personally learned, no doubt be sold in that market, or cut up for sausages. Firmly convinced that all this diseased meat was unfit for human food, I sought for some official all over the market, and in the clerk's office, but I could find none; consequently, to the desecration of the Sabbath was added the preparation of diseased food for the people, without any chance of the merited chastisement being inflicted. Such a system must act as a premium to knavery.

"So far as I have been able to ascertain, the slaughter-houses in the New Cattle Market are exempt from inspection: the clerk of the market so informed me, and he added that he regarded those establishments as private, inasmuch as though the buildings formed part of the public market, they were let to private individuals. If such be the fact, and I believe it practically is so, a premium is offered to sending diseased beasts to the cattle market; for the inspection of live stock being lax, and the slaughter-house exempt from supervision, the greatest facility is offered for disposing of diseased beasts and preparing their carcases for the butcher, with all those arts of trimming, dressing, and polishing, which are well known to veil appearances of disease, so as to beguile the inexpert, to facilitate a commercial fraud, and introduce the seeds of disease, and not unfrequently actual poison, into the unhappy individuals who unconsciously partake of the meat for the sustenance of their lives."

Again:—

"On Saturday afternoon, the 22d inst., I visited Newgate Market. The establishments of the large salesmen were all closed, and at numerous little shops meat was being sold, chiefly by men of disreputable appearance, to poor persons. The quantity of diseased meat, most unquestionably unfit for human food, was very large; what I saw in half an hour would have laden a single-horse cart: among other specimens, I saw, at the back of a little dark shop, a very thin, pale fore-quarter of beef, extensively ecchymosed, for which I was asked three pence per pound; in another place a saddle of mutton, the muscle of which was pale and pappy, the scanty fat, moist, and deeply tinged with the characteristic yellow of bile; many legs of mutton and huge pieces of beef were either in an advanced stage of putrefaction or bore unmistakable marks of organic disease. I sought about the market for officers, but I only found the beadle in his closet; the meat-inspectors were nowhere to be found, and I reluctantly made my way to the meeting-room of the Medical Society of London, to relate the scene of filth, fraud, and negligence which I had witnessed."

His discussion of the second and third propositions of his list need not occupy us here, although it is well sustained, and is sufficiently suggestive, as to the inadequacy of the inspection-laws complained of, to afford valuable hints to the municipal solons on this side of the ocean. Passing as well over them as over the first part of the fourth category, which relates solely to the prevention, by quarantine enactments, of the threatened invasion of the *murrain* or contagious typhus, through the importation, from the continent, of infected cattle or of infected raw-hides, we conclude our quotations with the paragraphs which treat especially upon the question of the effect of diseased meat upon those who use it in their food.

"A second no less urgent call for effective legislative enactments is the vast amount of diseased meat consumed for food by the population. Here it becomes of first importance to consider what is the effect on man of eating meat from an animal that has died or been killed with disease. Numerous authorities attest* that such alimentation may be and often is productive of the most

* "*D. Meier*, in Archiv für Thierheilkunde von der Gesellschaft Schweizerischer Thierärzte, Band xii. heft, 2, s. 148. *Albert*, k. 6. Landgerichtsarzt zu Enendorff Henkes Zeitschrift für Staatsarzneikunde, 22r. Jahrg, 1842, 3s, heft, s. 185. Vesicular Disease contracted from Sheep, by *George Burrows*, M.D., F.R.S., in *Med. Times and Gazette*, and *Veterinarian*, 1856. On the Production of Tænia considered in relation to public Hygiene, by Dr. *Riecke*, of Nordhausen, in Henle's Zeitschrift, *Edinburg Med. Jour.*, October, 1857. 'Case of Tape-worm occurring in connection with the eating of measled pork,' communicated by Dr. *W. T. Gairdner* to Medico-Chirurgical Society of Edinburgh. Clinical observations of effects of eating measley pork and unsound meat, by Dr. *Gibbon*, Dr. *Challice*, and others, in *Report on Unwholesome Meat*, by Committee of Metropolitan Association of Medical Officers of Health. Ueber das Fleisch der Schlachtbaren Hausthiere in Gewerblicher und Sanitäts-polizeilischer Beziehung. Ein Handbuch von Veterinair Assessor Hildebrandt. Magdeburg, 1855, s. 144. On the consignment of Sick Cattle to the Butcher, by Mr. Hosburgh, of Dalkeith, *Vet.*, 1842. Eduardo Turchetti in Archiv für Thierheilkunde, Band xii. heft, 1, Zurich, 1843, and Gaz. Méd. de Paris, 6th Aug., 1842. Hering Leistungen in der Thierarzneikunde, p. 66. Vermeintliche Brechweinsteinvergiftung bei Menschen in Folge des Genusses von Fleische eines Zuvor mit Brechweinstein behandelten Ochsen-Mitgeheilt vom Herausgeber in Centraltzeitung für die Gesammte, Veterinärmedizin, herausgegeben von Dr. Johann Martin Kreutzer; Vierter Jahrgang; Erlangen, 10 Mai, 1854, s. 74, und Forts. Umschau auf dem Gebiete der Staatsveterinärmedizin von Dr. Johann Martin Kreutzer in Centralzeitung, supra cit. Beitrag zur Erledigung der Frage, ob der genuss des Fleisches Milzbrandkranker Thiere, schadlich sei oder nicht. Dr. Rosenthal, in Caspar's Viertaljahrschrift für gerichtliche und öffentliche Medizin, 1854, Sweites Heft.

"Canstatt's 'Jahresbericht über die Leistungen in des Thierheilkunde' in Jahre, 1853, s. 65, und in Jahre, 1854, p. 61. Dr. A. Neuman, 'Ueber den genuss dess baneful results, even unto speedy death. The fact that many persons have often subsisted on animal food of the worst kind, is no more an argument against the injuriousness of such alimentation than would be a plea for the harmlessness of a cholera or intermittent-fever atmosphere, founded on the fact that a large number of persons may breathe it without apparent suffering. The fair presumption is, that from impure materials the sustenance of the human body cannot be derived without risk; and, accordingly, experience teaches, that although by the marvellous organic and functional provisions of the animal economy, injurious influences from without are, in great measure, counteracted, yet impure air, water, and solid aliment cannot be introduced into the system without weakening the vital powers, and often without the most disastrous immediate results. Moreover, it is most fair to argue that the number of cases of illness referable to the eating of diseased meat is even much greater than that recorded in the annals of science, it being impossible in very many instances to trace back the causes of a disease and to ascertain what kind of animal food has been partaken of.

"It is perfectly certain that most sausage-makers use up the diseased parts, which even the poor would not buy; the frequenters of eating-houses, particularly in some neighborhoods, are largely supplied with the best-looking parts of diseased beasts; and even in good society no guarantee exists against such occurrence, beyond the honesty of individual purveyors. And be it not imagined that because the hind-quarters of a beast that has died with typhus, or the fore-quarters of a fine fat young cow that has died with puerperal fever, may chance to look well, therefore they are harmless as food. The poisoned blood has circulated through every tissue, and, besides its dose of animal poison, it has been the carrier of the medicinal substances that may have been administered to the beast before death. I have supplied reference to a case of poisoning in man from eating the flesh of an ox that had been treated with tartarized antimony; and Professor Macadam, of Edinburgh, has experimentally proved that strychnine is discoverable, and in notable quantity, in the tissues of a dog that had been fed on the flesh of a horse killed with strychnine. It is a fact, which I can produce in evidence, that a cow-keeper at Chelsea would not let a veterinary surgeon continue to give sulphuric ether to one of his sick cows because it had been smelled in the milk by one of his customers. Other drugs were substituted, health did not return, and in the regular course, after having supplied its diseased and medicated milk, the beast was sent to the slaughterer, and thence partly to the sausage-maker and partly to the butcher!

"An aspect of the case not to be lost sight of is, that this traffic in diseased

---

Fleisches Kranker Thiere,' in Het. Repertorium Tydschrift voor de Geneeskunde in al haren Omvang, Leiden, 1853. Professor John Gamgee on 'Unwholesome Meat,' in Scotsman, 28th Feb., 1857. Congrossi Acta Erudit. an. 1713–14, Malad. Epiz. p. 125. Anleitung den Gesundheitszustand und die Krankheiten der Schlachtbaren Hausthiere im lebenden wie geschlachteten zustande zu erkennen von Königl. Regierungs. Departements, Thierarzt zu Breslau, F. Grüll, Breslau, 1848. Verheren, 'Sur la vente de la chair des animaux atteints de certains maladies.'—Rapport fait à l'Académie Royale de Médecine de Belgique, *vide* Recueil de Méd. Vét. 1847, p. 851, *et seq.*"

beasts is a most dishonest one; that the majority of those who are engaged in it know it to be such, and employ every art to deceive the population, the poor part of which most grievously suffers. Assuming that every one will admit the evil is one of the most enormous magnitude and seriousness, I shall now proceed to consider the principles on which legislative enactments should be based to arrest it.

"In the first place it is important to establish that the flesh of animals dying from or with disease is not invariably prejudicial to man: in many cases its injurious effects are extreme, in others they do not appear. Hence the necessity of defining the line of demarcation; because it is a great *desideratum* to lessen the loss from disease to cattle-breeders and dealers, and to allow a cheap article of food for the population, so long as such advantages are not attended with counterbalancing evils. It is a curious fact, of great practical moment to be known, that the extent in which the flesh of diseased animals is injurious to man is not in exact proportion to the degree in which the disease is fatal to themselves. Thus: whereas, oxen affected with pleuro-pneumonia frequently die in a few days, their carcases may be eaten—the actually diseased parts excepted—with comparative impunity; but the flesh of malignant anthrax, of the cynanche maligna of pigs, of puerperal fever, and gangrenous erysipelas, is capable of producing the most disastrous consequences, if partaken of as human food. Moreover, all parasitic affections affecting the edible parts of animals are most serious; because scientific experience has amply demonstrated that men partaking of them become themselves infected with worms; the cœnurus cerebri of the sheep, and the cysticercus cellulosæ of pigs, develop into the tænia in the dog and man. These facts render absolutely necessary the inspection of live and dead meat markets by persons endowed with the knowledge, which only experience, obtained carefully with the lights of science, is capable of imparting."

The second letter of Mr. Gamgee is supplemental in its character; and, although ably and efficiently seconding his first appeal, embodies no new facts of sufficient special importance to justify a further notice of its contents.

These pamphlets, and the discussion which grew out of their publication and still continues in the journals of the day, cannot fail to stimulate—what is always beneficial—a great deal of well-directed and intelligent inquiry as well as strictly scientific investigation, whatever the result upon the legislative action of the country. The hygienic question may be regarded as only just begun, notwithstanding the labors of Soumille Renault, Greenhow and others, on both sides of the British Channel. We hope to be able ere long to approach it once more with data more definite and abundant than has yet been placed within our reach, above all in its relations with American experience.

